# Prevalence of *Porphyromonas gingivalis* Four *rag* Locus Genotypes in Patients of Orthodontic Gingivitis and Periodontitis

**DOI:** 10.1371/journal.pone.0061028

**Published:** 2013-04-04

**Authors:** Yi Liu, Yujie Zhang, Lili Wang, Yang Guo, Shuiqing Xiao

**Affiliations:** 1 Pediatric Research Institute, Qilu Children's Hospital of Shandong University, Ji'nan, Shandong, China; 2 Department of Orthodontic, Jinan Stomatological Hospital, Jinan, Shandong, China; 3 Clinical Laboratory, Jinan Central Hospital of Shandong University, Jinan, Shandong, China; Jagiellonian University, Poland

## Abstract

*Porphyromonas gingivalis* is considered as a major etiological agent in periodontal diseases and implied to result in gingival inflammation under orthodontic appliance. *rag* locus is a pathogenicity island found in *Porphyromonas gingivalis.* Four *rag* locus variants are different in pathogenicity of *Porphyromonas gingivalis*. Moreover, there are different racial and geographic differences in distribution of *rag* locus genotypes. In this study, we assessed the prevalence of *Porphyromonas gingivalis* and *rag* locus genotypes in 102 gingival crevicular fluid samples from 57 cases of gingivitis patients with orthodontic appliances, 25 cases of periodontitis patients and 20 cases of periodontally healthy people through a 16S rRNA-based PCR and a multiplex PCR. The correlations between *Porphyromona.gingivalis*/*rag* locus and clinical indices were analyzed. The prevalence of *Porphyromonas gingivalis* and *rag* locus genes in periodontitis group was the highest among three groups and higher in orthodontic gingivitis than healthy people (p<0.01). An obviously positive correlation was observed between the prevalence of *Porphyromonas gingivalis*/*rag* locus and gingival index. *rag*-3 and *rag*-4 were the predominant genotypes in the patients of orthodontic gingivitis and mild-to-moderate periodontitis in Shandong. *Porphyromonas.gingivalis* carrying *rag-1* has the strong virulence and could be associated with severe periodontitis.

## Introduction

Malocclusion is one of the most common oral-maxillofacial diseases that bring some negative effects on facial aesthetics, oral physical function and health as well. In China, morbidity of malocclusion in teenagers is as high as 67.82% [1]. Orthodontic treatment is currently the preferred and most common method for the reason of solving above the problems, but it also holds some potentials of harming teeth and periodontal tissues due to plaque accumulation and gingival inflammation that are induced by the changes of oral internal environment after wearing fixed orthodontic appliance, therefore lead to changing of host physiology and the composition of the oral microflora [2], [3]. The inflammatory reaction of gingival tissue can often be detected in patients wearing fixed orthodontic appliances. The overall morbidity of gingivitis was higher as 56.8% and 34.4% in adolescent group and adult group respectively during fixed orthodontic treatment in China [4]. Primary pathogenic microorganisms strongly implicated in gingival inflammation and posterior periodontal destruction, such as *Porphyromonas gingivalis*, *Prevotella intermedia/nigrescens*, *Aggregatibacter actinomycetemcomitans*, *Tannerella forsythia*, *Treponema denticola*, and *Fusobacterium* species have been found elevated in patients after bracket placement [5]–[7]. Our previous research showed that the percentage of *bacilli*, esp. *Porphyromonas gingivalis*, *Aggregatibacter actinomycetemcomitans*, *Fusobacterium nucleatum* increased significantly after wearing orthodontic appliance and the increase of those pathogens was significantly related with the development of gingivitis in orthodontic treatment [8]–[9].

*Porphyromonas gingivalis* is a gram-negative oral anaerobe and considered as a major etiological agent in periodontal diseases by producing a number of virulence factors and extracellular proteases, such as lipopolysaccharide, capsule, gingipain, fimbria and so on, resulting in the destruction of periodontal tissues [10]–[13]. The pathogenicity of *P.gingivalis* has been investigated in a variety of experimental animal models, such as rat [14]–[15], mouse [16]–[17], rabbit [18], drosophila [19], and cell models [20]–[23], showing complicated mechanisms of *P.gingivalis*-host interactions in development of periodontal diseases. Three gingipains referred to be the important virulence factors, Arg-x-specific proteinase and adhesins (RgpA), Arg-x-specific proteinase (RgpB), and a Lys-x-specific proteinase and adhesins (Kgp) have been well known and studied in details with properties of activating and/or degrading a wide range of host proteins through different mechanisms [20]–[23].

Pathogenicity island is a large unstable chromosome DNA region encoding virulence determinants of pathogenic bacteria and was first described in human pathogens of the species *Escherichia coli* by Hacker et al.[24]. The island has been also detected in other pathogens, such as *Klebsiella pneumoniae*, *Enterobacter aerogenes*, and *Citrobacter koseri* isolates with high conservative property among these species and is in association with the yersiniabactin determinant [25]. In 1999, Curtis et al [26] found a novel pathogenicity island in a proportion of *P.gingivalis* strains named *rag* locus and it was more frequently detected in deep periodontal pockets in periodontal patients. It was reported that the *rag* locus of *P.gingivalis* might arise from *Bacteriodes* via horizontal gene transfer and encodes *RagA* and *RagB*. *RagA* is a 115-kDa TonB-dependent outer membrane receptor, and *RagB* is a 55-kDa lipoprotein constituting an immunodominant outer antigen. Both proteins of *RagA* and *RagB* constitute a membrane transporter system. Further study demonstrated that four *rag* locus variants with different pathogenicity were detected from clinical isolates of *P.gingivalis* [26]–[28]. A significant correlation was observed between prevalence of *rag-1* allele and a highly virulent phenotype in a murine model of soft tissue destruction [29]. In addition, there are different racial and geographic differences in distribution of *rag* locus genotypes [28]. Wang et al.[30] investigated the distribution of *rag* genotypes in chronic periodontitis patients in Northeast of China and found *P.gingivalis* carrying *rag-1*, *rag-3* was more predominant in chronic periodontitis so that might be associated with the development of pediodontitis.

There have been reports about the association between prevalence of *P.gingivalis* and gingival inflammation during orthodontic treatment [31]–[35]. Our previous research showed that the prevalence of *P. gingivalis* was totally higher as 40.62% two months after orthodontics detected by using traditional anaerobic culture [9]. While there have been no investigations about the correlation between *P.gingivalis rag* locus and periodontal health status in orthodontic gingivitis patients. Therefore, we assessed the prevalence of *P.gingivalis* and *rag* locus genotypes in gingival crevicular fluid samples from the gingivitis lesions of orthodontic patients and compared them with periodontitis patients as well as periodontally healthy people who showed healthy periodontal tissues before wearing orthodontic appliances.

## Materials and Methods

### Subjects

The study subjects consisted of three groups who visited Jinan stomatological hospital for orthodontic or periodontitis treatment from 2010 to 2011. Of three groups, orthodontic group (OG) included 57 patients, 38 females and 19 males, aged between 10 and 30 years (mean 16.3) who got gingival inflammation during orthodontic treatment; control group (CG) contained 20 periodontally healthy people, 12 females and 8 males, aged between 10 and 30 years (mean 16.05) before orthodontic treatment; periodontitis group (PG) was composed of 25 periodontitis patients, 10 females and 15 males, aged from 20 to 60 years (mean 25) who came to hospital for periodontitis treatment. The patients who are having any systemic diseases, antibiotics therapy within the last 3 months and pregnant or lactating females were excluded.

### Ethics statement

This work was approved by the Medical Ethics Committee of the Jinan Stomatological Hospital. All patients or their parents gave their verbal followed by written informed consent before the examination was performed. The relevant regulations and institutional polices were followed strictly.

### Bacteria strains

The reference strains of *P.gingivalis* ATCC33277, *F.nucleatum* ATCC25586 and *A.actinomycetemcomitans* ATCC29522 were from the West-China Dental School of Sichun University and *P.gingivalis* W83 was from Beijing Oral Research Institute of Capital Medical University.

### Clinical examination

We selected the gingivitis or periodontal sites that exhibited the deepest pocket depth of every subject. The clinical parameters included gingival index (GI), plaque index (PI), sulcus bleeding index (SBI) and probing depth (PD) of each person were examined and recorded. All clinical examinations were carried out by the same dentist.

### Sample collection and DNA extraction

Gingival crevicular fluid (GCF) was obtained from the two deepest periodontal pockets in the maxilla according to Rüdin et al [36]. In brief, before collecting, saline solution was used to rinse out food debris and then each site was cleaned by cotton rolls. Sterile filter paper strips were placed for 30 seconds into the packet until a minimum of resistance. The paper points were placed into a sterile microcentrifuge tube containing 0.5 ml of 1×PBS immediately. The tubes were mixed thoroughly and stored at −20°C until analyzed. The bacterial DNA was extracted by the boiling method [37]. In short, a 10 µl aliquot of each stored sample was added to 10 µl of 2 ×lysis buffer (2 mM EDTA, 1% X-100). The mixture was boiled for 10 minutes and then placed on ice. The supernatant was used as the template for PCR amplification.

### The 16S rRNA-based PCR and multiplex PCR amplification

The 16S rRNA gene specific primers were used to determine the prevalence of *P.gingivalis* in GCF, while four different *rag* locus variants primers were utilized to amplify the rag locus variants genes from GCF samples containing *P.gingivalis*. The 16S rRNA-based PCR was first performed on DNA extracts from GCF samples using primers of 16S rRNA-F (5′-AGG CAG CTT GCC ATA CTG CG-3′) and 16S rRNA-R (5′-ACT GTT AGC AAC TAC CGA TGT-3′) that amplify a 404-bp region of the 16S rRNA gene[38]. The specificity of this PCR was confirmed by sequencing and amplifying *P.gingivalis* ATCC33277, W83, as well as unrelated pathogens *F.nucleatum* ATCC25586 and *A.actinomycetemcomitans* ATCC29522. Then the multiplex PCR was utilized to amplify *rag* locus genes from the positive *P.gingivalis* samples. Amplification reaction was run in a Tetrad Thermal Cycler (MJ Research, South San Francisco, USA) in a 25 µl reaction mixture containing 4.5 µl 10×PCR buffer (100 mM Tris-HCl, 500 mM KCl, and 15 mM MgCl2), 0.25 mM of each deoxynucleoside triphosphate (dNTP), 10 µM of each primers, 5 µl of DNA extracts from GCF samples, and 1.5 units of Taq DNA polymerase (Transgen Biotech, Beijing) for 5 min at 94°C and 33 cycles, with each cycle consisting of denaturation at 94°C for 30 sec, annealing at 57°C for 30 sec, extension at 72°C for 1 min, and final extension for 10 min. Nucleotide sequences of the forward and reverse primers for *rag* locus genes were listed in Table 1 [39].

**Table 1:** Primers of *rag* locus genotypes used for PCR.

Primers	Sequences(5′→3′)	Sizes (bp)
*rag-1F*	5′-CGCGACCCCGAAGGAAAAGATT-3′	628
*rag-1R*	5′-CACGGCTCACATAAAGAACGCT-3′	
*rag-2F*	5′-GCTTTGCCGCTTGTGACTTGG-3′	979
*rag-2R*	5′-CCACCGTCACCGTTCACCTT-3′	
*rag-3F*	5′-CCGGAAGATAAGGCCAAGAAAGA-3′	423
*rag-3R*	5′-ACGCCAATTCGCCAAAGCT-3′	
*rag-4F*	5′-CCGGATGGAAGTGATGAACAGA-3′	739
*rag-4R*	5′-CGCGGTAAACCTCAGCAAATT-3′	

The amplified PCR products were then electrophoreses on 1.5% agarose gel in Tris-acetate buffer (40 mM Tris acetate, 1 mM EDTA, pH 8.0). The products were visualized with ethidium bromide by UV transillumination.

### Statistical analysis

The differences in the prevalence of *rag* locus genes were analyzed using the Chi square test. The Spearman Correlation Test was utilized to analyze the correlation between prevalence of *P.gingivalis/rag* locus genes and clinical indices in three research groups. All statistical analyses were done by using a statistical software package (SPSS for Windows 13.0).

## Results

### Detection and confirmation of 16S rRNA-based PCR for *P.gingivalis*

The reference stains were first amplified by the 16S rRNA-based PCR to evaluate the specificity of it. The positive product appeared only from *P.gingivalis* ATCC33277 and W83, not from *F.nucleatum* ATCC25586 and *A.actinomycetemcomitans* ATCC29522.

Sixty-five *P.gingivalis* was detected in 65 (63.73%) cases of GCF samples from 102 cases of three groups, thirty-five (61.40%) from orthodontic group (OG), Seven (35%) from control group (CG), and 23 (92%) from periodontitis group (PG). Prevalence of *P.gingivalis* was found significantly different in three groups: *P.gingivalis* was the highest prevalence in PG (P<0.01) and higher level in OG than CG (P<0.05) (Table 2). Of 65 *P.gingivalis* positive samples, 10 were randomly sequenced by a 3730 DNA sequencer in Invitrogen Company (Invitrogen, Shanghai) and confirmed the validity of the 16S rRNA-based PCR for clinical GCF samples (date were not showed).

**Table 2:** Prevalence of P.gingivalis among three groups.

Groups	Cases (n)	Prevalence of P.gingivalis	
		P.gingivalis counts	P.gingivalis(%)
OG	57	35	61.40*
CG	20	7	35.00
PG	25	23	92.00**
Total	102	65	63.73

** P<0.01 between PG and CG; * P<0.05 between OG and CG (Chi-squared test).

The correlation of patients' age and prevalence of *P.gingivalis* was analyzed. The average age of patients with positive *P.gingivalis* in GCF was 25.54 years, while patients with negative *P.gingivalis* was 16.19 years, and there was statistical difference between the patients' ages of both positive and negative *P.gingivalis* (P<0.05).

### Multiplex-PCR amplification of *rag* locus genes

Multiplex-PCR was firstly used to detect *rag* locus genes from the high virulent *P.gingivalis* W83 and low virulent *P.gingivalis* ATCC33277. It showed that *rag-1* gene was amplified from *P.gingivalis* W83 and *rag-4* was from ATCC33277, which were consistent with previous documents [40], [41].

**Figure 1:**
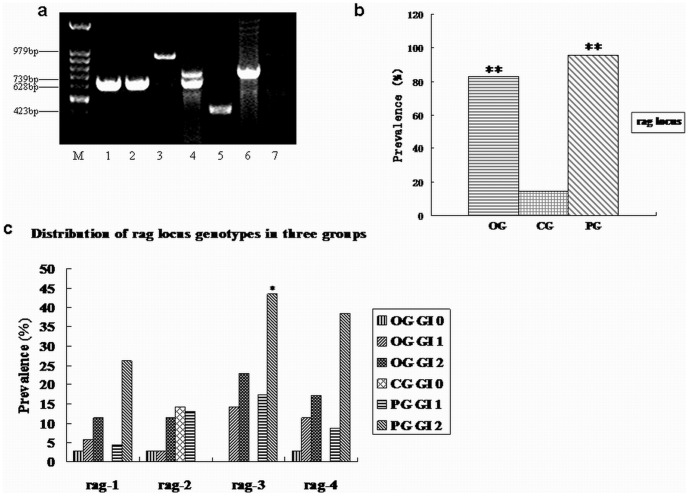
Detection and distribution of *rag* locus genes. a. Detection of rag locus genes in clinical GCF samples. M DNA Marker; Lane1 positive control of *P.gingivalis* W83; Lane 2–7 clinical GCF samples, showing *rag-1* (lane 2), *rag-2* (lane3), *rag-1* combined with *rag-4* (lane4), *rag-3* (lane5), *rag-4* (lane6), and negative (lane 7). b. The prevalence of *rag* locus genes in clinical GCF samples of three groups. ** P<0.01 between OG/PG and CG (Chi-squared test). c. Distribution of four *rag* locus genes among three groups.

There were 52 (80%) positive *rag* locus genes detected from the 65 GCF samples which were *P.gingivalis* positive, twenty-nine (82.86%) from those in OG, 22 (95.65%) from those in PG, only one case (14.29%) from healthy people. The prevalence of *rag* locus was significantly higher in PG and OG than in CG (P<0.01), while no significant difference between PG and OG (P>0.05) (Fig. 1).

Among *P.gingivalis*-positive GCF samples from three groups, the most prevalent rag gene was rag-3 (51.92%), followed by rag-4(38.26%) and rag-1(26.92%). While in those of PG, rag-3 (60.87%), rag-4 (39.13%), rag-1(30.43%) were much higher than those in CG (only rag-2 positive); Similar to those of periodontitis group, in those of OG, the proportion of *rag* genotypes were: *rag-3* 44.83%, *rag-4* 37.93%, *rag-2* 20.69%; but in CG: only one case of *rag-2* (14.29%) was detected.

### Correlation of *rag* locus genes and clinical indices

**Figure 2:**
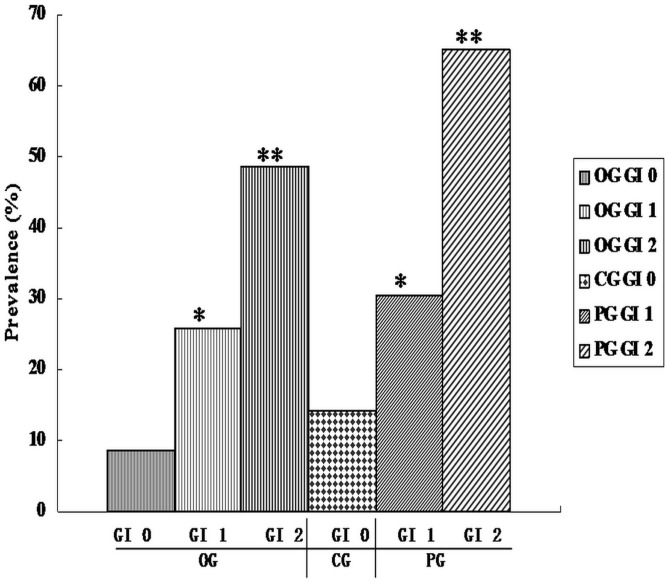
Correlation of rag locus genes and gingival indices (GI). ** P<0.01 between GI 2 and GI 0; * P<0.05 between GI 1 and GI 0, ^#^ P<0.05 between GI 2 and GI 1 in PG (Chi-squared test).

It was noticed that clinical indices were all higher in OG and PG than CG (P<0.05). The prevalence of *rag* locus genes, except rag-2, elevated directly with increases of GI values in both OG and PG. The significant positive correlation between *rag* locus and GI was showed by using Spearman Correlation Test (P<0.01) (Fig. 2). However, there were no statistical difference between *rag* locus and PD/PI/SBI, while we detected the increase of *rag* locus in OG and PG with higher GI and higher PD/PI/SBI. *rag-1* often appeared from the deeper periodontal pocket with higher PD/PI/SBI values and 10/14 of *rag-1* positive cases accompanied with *rag-3* and/or *rag-4*. Interestingly, an exception was found that one case of *rag-1* gene was from a patient of GI 0 lever in orthodontic group, so further research would be done for the possible variation of *rag-1* gene. The prevalence of four *rag* locus genes among three groups showed in table 3.

**Table 3:** Prevalence of *rag* locus under different gingival index (GI) among three groups.

Groups	GI (n)	rag locus(%)	rag-1 (%)	rag-2 (%)	rag-3 (%)	rag-4 (%)
OG	0 (16)	8.57	2.86	2.86	0	2.86
	1 (20)	25.72*	5.72	2.86	14.29	11.43
	2 (21)	48.57**	11.43	11.43	22.86	17.14
CG	0 (20)	14.29	0	14.29	0	0
PG	1 (10)	30.43*	4.35	13.04	17.39	8.70
	2 (15)	65.22**	26.09	0	43.48#	38.46

** P<0.01 between GI 2 and GI 0; * P<0.05 between GI 1 and GI 0,

# P<0.05 between GI 1 and GI 0 (Chi-squared test).

## Discussion

*P.gingivalis* has been known to be a risk factor for periodontal diseases though the exact roles of it in the initiation and progression of the oral diseases remain unclear. Mouse model tests have indicated difference in the virulence of *P.gingivalis* with and without *rag* locus [28], [29]. Shi et al. mutated *rag* locus genes in *P.gingivalis* by using an allele replacement strategy and clearly showed that inactivation of the rag locus reduced the virulence of *P.gingivalis* in a mouse model of soft tissue destruction [29]. In a collection of 168 isolates of *P.gingivalis* from western European countries, including the Netherlands, Romania, Sweden, the United Kindom, Kenya and Germany, Hall et al. found different prevalence and geographic differences in distribution of four *rag* alleles [28]. In this case, we detected prevalence of *P.gingivalis* and *rag* locus genotypes in local patients of orthodontic gingivitis, periodontitis and periodontally healthy people to evaluate the distribution of *P.gingivalis* and predominant genotypes of *rag* locus in different periodontal health statuses, then further deduce the pathogenicity of *P.gingivalis* carrying different rag locus during orthodontic treatment.

In periodontal disease, gingival crevicular fluid (GCF) is an inflammatory exudate. GCF contains substances from supra-and subgingival located bacteria. Analysis of microflora in GCF becomes more and more important in diagnosis and therapy of periodontal diseases. There are a large number of periodontopathic bacteria including *P.gingivalis* in GCF [42]. Considering the method of filter paper strips is recommended to collect the microflora from GCF for microbiological analysis in dental practice [35]. With sterile filter paper strips, we collected GCF samples divided into orthodontic gingivitis, periodontally healthy control and periodontitis groups from 102 patients to investigate the prevalence of *P.gingivalis* in above three groups. The occurrence of *P.gingivalis* was 61.40% in OG, 35% in CG, and 92% in PG, respectively. There was a statistically higher prevalence of *P.gingivalis* in PG followed by OG than CG. Furthermore, a significantly strong positive correlation was observed between the prevalence of *P.gingivalis* and GI by Spearman Correlation Test (P<0.01), which was consistent with previous reports [31]–[33], [43]. While once wearing fixed appliance, oral hygiene will turns bad if teeth cleaning can not be paid special attention, as a result, dental plaque will accumulate and gingival inflammation will happen. By then, one side, anaerobic environment will be created due to swollen gum, deeper gingival sulcus, and pseudo periodontal pocket; on the other side, the gum will be susceptible to bleeding and therefore it will be more conductive for periodontal anaerobic *P.gingivalis* to survive. *P.gingivalis* may play a similar role in orthodontic gingivitis and periodontitis.

*P.gingivalis* has been reported to be related with adult periodontitis [10], [12]. In this study, we analyzed correlation of patients' age and occurrence of *P.gingivalis* and found the age of both *P.gingivalis* positive and negative was statistically different; implying the prevalence of *P.gingivalis* may increase as patients'age increased.

In order to further explore whether the *P.gingivalis rag* locus was associated with gingival inflammation under orthodontic appliance,we detected the distribution of *rag* locus in three groups and discovered 52 (80%) of positive *rag* locus genes from 65 *P.gingivalis*-positive GCFs. The prevalence of *rag* locus was higher in those of PG and OG than those of CG. The *P.gingivalis* without *rag* locus was mostly detected from periodontally healthy control and GI 0 level OG patients, demonstrating that they present the avirulent or weak virulence genotype of *P.gingivalis*. A clear positive correlation was indicated between the gingival index and *rag* locus genes, implying *rag* locus genes may play a pathogenic and similar role in the development of gum inflammation during orthodontic in comparison with periodontitis.

The prevalence of *rag-3* (27 cases) was the most detected followed by *rag-4* (20 cases) and *rag-1* (14 cases); the lowest occurrence was *rag-2* (10 cases) with lower GI and PD/PI/SBI values, showing the *rag-3* and *rag-4* locus genes might be the predominant genotypes in the patients of orthodontic gingivitis and mild-to-moderate periodontitis in the populations of Shandong region. Besides, we found *rag-1* was detected from 14 cases, mostly with higher GI and PD/PI/SBI, and often combined with *rag-3* and/or *rag-4,* suggesting the *P.gingivalis* carrying *rag-1* is the strong virulent genotype and can be closely associated with severe periodontitis, which is consistent with Hanley et al[44].

In summary, *P.gingivalis* carrying *rag-3*, *rag-4* locus is one of the risk factors that are responsible for gingivitis during orthodontic treatment. Thus monitoring *P.gingivalis* is highly recommended following the placement of orthodontic appliances. In addition, appropriate oral hygiene is necessary to reduce invasion of pathogens and exerts a beneficial effect to oral tissues.
